# A randomized phase II/III trial of rosuvastatin with neoadjuvant chemo-radiation in patients with locally advanced rectal cancer

**DOI:** 10.3389/fonc.2025.1450602

**Published:** 2025-03-19

**Authors:** Prachi S. Patil, Avanish Saklani, Naveena A. N. Kumar, Ashwin De’Souza, Rahul Krishnatry, Snehal Khanvilkar, Mufaddal Kazi, Reena Engineer, Vikas Ostwal, Anant Ramaswamy, Munita Bal, Priya Ranganathan, Ekta Gupta, Sanjeev Galande

**Affiliations:** ^1^ Department of Digestive Diseases and Clinical Nutrition, Tata Memorial Hospital, Homi Bhabha National Institute, Mumbai, India; ^2^ Department of Surgical Oncology, Tata Memorial Hospital, Homi Bhabha National Institute, Mumbai, India; ^3^ Department of Surgical Oncology, Manipal Comprehensive Cancer Care Center, Kasturba Medical College, Manipal, Manipal Academy of Higher Education, Manipal, Karnataka, India; ^4^ Department of Radiation Oncology, Tata Memorial Hospital, Homi Bhabha National Institute, Mumbai, India; ^5^ Tata Memorial Hospital, Homi Bhabha National Institute, Mumbai, India; ^6^ Department of Medical Oncology, Tata Memorial Hospital, Homi Bhabha national Institute, Mumbai, India; ^7^ Department of Pathology, Tata Memorial Hospital, Homi Bhabha National Institute, Mumbai, India; ^8^ Department of Anaesthesiology, Tata Memorial Hospital, Homi Bhabha National Institute, Mumbai, India; ^9^ Laboratory of Chromatin Biology and Epigenetics, Department of Biology, Indian Institute of Science Education and Research, Pune, India; ^10^ Centre of Excellence in Epigenetics, Department of Life Sciences, Shiv Nadar Institution of Eminence, Delhi, India

**Keywords:** rosuvastatin, pathological complete response, neoadjuvant chemoradiation, rectal cancer, drug repurposing

## Abstract

**Aim:**

Statins have been shown to improve the possibility of a pathological complete response (pCR) in patients with locally advanced rectal cancer when given in combination with neo-adjuvant chemo-radiation (NACTRT) in observational studies. The primary objective of this phase II randomized controlled trial (RCT) is to determine the impact of rosuvastatin in improving pCR rates in patients with locally advanced rectal cancer who are undergoing NACTRT. The secondary objectives are to compare adverse events, postoperative morbidity and mortality, disease-free survival (DFS), and overall survival in the two arms and to identify potential prognostic and predictive factors determining outcomes. If the study is positive, we plan to proceed to a phase III RCT with 3-year DFS as the primary endpoint.

**Methods:**

This is a prospective, randomized, open-label phase II/III study. The phase II study has a sample size of 316 patients (158 in each arm) to be accrued over 3 years to have 288 evaluable patients. The standard arm will receive NACTRT while the intervention group will receive 20 mg rosuvastatin orally once daily along with NACTRT for 6 weeks followed by rosuvastatin alone for 6–10 weeks until surgery. All patients will be reviewed after repeat imaging by a multidisciplinary tumor board at 12–16 weeks after starting NACTRT and operable patients will be planned for surgery. The pathological response rate, tumor regression grade (TRG), and post-surgical complications will be recorded.

**Conclusion:**

The addition of rosuvastatin to NACTRT may improve the oncological outcomes by increasing the likelihood of pCR in patients with locally advanced rectal cancer undergoing NACTRT. This would be a low-cost, low-risk intervention that could potentially lead to the refinement of strategies, such as “watch and wait”, in a select subgroup of patients.

**Clinical trial registration:**

Clinical Trials Registry of India, identifier CTRI/2018/11/016459.

## 1 Introduction

### 1.1 Background

Colorectal cancer (CRC) is the third most common cancer in men (746,000 cases, 10.0% of the total) and the second in women (614,000 cases, 9.2% of the total) worldwide (1). In India, CRC is the third most common cancer (1, 2). In India, the annual incidence rates (AARs) for colon cancer and rectal cancer in men are 4.4 and 4.1 per 100,000, respectively (3).

The standard treatment for locally advanced rectal cancer (stage T3 and above and N1 and above) is neo-adjuvant chemo-radiation (NACTRT) followed by surgery and adjuvant chemotherapy (4, 5). The response to NACTRT is assessed by radiology and histopathology (4, 5). A pathological assessment is considered the gold standard tool for assessment of response (6). The prevalence of a pathological complete response (pCR)—defined as no residual cancer found on histological examination of the total mesorectal excision (TME) specimen—varies from 15%–27% following NACTRT (7). In addition to a favorable prognosis, if a complete response is noted at reassessment, a pCR permits a potentially rectal-preserving watch-and-wait strategy. Recent studies indicate a higher chance for a wait-and-watch strategy given the nearly double pCR rates previously reported in the UNICANCER-PRODIGE 23 (28% vs. 12%), RAPIDO (28% vs 14%), and STELLAR trials (22% vs 12%) (45). The patients who achieve pCR after NACTRT have better long-term outcomes, less propensity to develop local and distant recurrence, and improved survival (8). In these patients, sphincter-preserving procedures or organ-preserving options, such as local excision of residual tumor (9) or omission of surgery altogether, have been evaluated (10–12). In a meta-analysis including 3,105 patients, Maas et al. demonstrated (i) that the 5-year crude disease-free survival (DFS) rate of 484 patients who achieved a pCR after NACTRT was 83%, compared with 66% for those who did not have a pCR (*p* < 0.0001) and (ii) that the 5-year distal metastasis-free survival rate was 89% in the pCR group and 75% in the non-pCR group (*p* < 0.0001) (13). Further, patients with a pCR in the RAPIDO trial were divided into two groups: experimental [EXP,120/423, short-course radiotherapy, chemotherapy, surgery, and total neoadjuvant therapy (TNT)] and standard-of-care treatment (STD, 57/398, CRT, surgery, postoperative chemotherapy depending on hospital policy). They reported the 5-year overall survival (OS) rate was 94% [95% confidence interval (95%CI)= 90–98] in the EXP group and 93% (95%CI= 87–100) in the STD group [hazard ratio (HR) =1.41, 95%CI =0.51–3.92; p = 0.50] (45).

Achieving a pCR following NACTRT is probably a prerequisite for improved outcomes in rectal cancer (7) using neo-adjuvant therapy. There have been multiple strategies suggested to improve pCR with mixed results including induction chemotherapy prior to and after NACTRT, delay in surgery after NACTRT, chemotherapy, and short-course radiotherapy. Recent total neoadjuvant (TN) trials have shown improved complete response rates using a combination of induction or consolidation chemotherapy with either long-course or short-course radiotherapy (14–17). A meta-analysis study for locally advanced rectal cancer was conducted to compare the efficacy of TN and NACTRT (38). The analysis considered six studies with 12,812 subjects for the meta-analysis and reported no significant difference in pCR and local relapse rate. Furthermore, distant metastasis was found to be better with TN than with NACTRT (14.3% vs. 20.4%), while the difference was not statistically significant [Odds ratio (OR)=0.84, 95%CI =0.31–2.27] (38). None of these trials were without toxicity related to treatment intensification. The addition of contact brachytherapy to NACTRT also increases complete response in small-sized tumors (18). If an increase in pCR rates can be achieved without increasing the treatment duration or adding toxicity, it would be worth exploring. One strategy that might improve the pCR is to add statins due to their anti-neoplastic effect along with NACTRT even though it is not an established strategy (19, 20). Few observational studies have suggested the benefit of statins in rectal cancer (19, 20) in terms of pCR, but pCR has been incidentally noted following NACTRT in patients receiving statins. The pCR rate was 25%–30% with statins compared to 15%–17% without statins (19, 20). In a phase II trial study wherein simvastatin was added along with chemoradiotherapy (CRT) and capecitabine in patients with locally advanced rectal cancer, the pCR rate was 18.9% (n=10) in the per-protocol analysis (46). Further, in an observational study, the authors reported that the disease-free survival did not vary (HR=0.98, CI=0.77–1.25, p=0.88) even if the patients were given statins along with CRT (47). However, there are no completed randomized controlled trials (RCTs) on the third-generation statin, rosuvastatin, in patients with locally advanced rectal cancer. Furthermore, an increasing amount of data suggests that obesity, dietary variables, and lipids directly contribute to the development of various cancerous tumors, which frequently result in treatment resistance and metastasis (44). Therefore, aggressive malignant tumors may become more vulnerable if statins are used as an adjuvant in anti-neoplastic treatment regimens. Mechanistically speaking, apoptosis, the suppression of proliferation via STAT3/SKP2 signaling, and the modification of the YAP/CD44 growth axis through YAP inactivation are the significant mechanisms behind statins’ anti-neoplastic effects (44). By downregulating TAZ, which is mediated by p53 transcriptional overexpression, statins have been demonstrated to impact the viability of cancer cells (44). According to a recent study, statins may cause epigenetic changes by inhibiting DNA methyltransferases (DNMT) while supporting CRC stem cell differentiation (44). These novel attributes of statins, however, which include focusing on the inhibition or degradation of carcinogenic proteins, offer novel perspectives of how statins work, whether they work in tandem with or independently of the cholesterol pathway.

The mechanisms of action of statins are manifold. Inhibition of hydroxymethylglutaryl- CoA (HMG-CoA) reductase by statins leads not only to a decrease in circulating low-density lipoprotein (LDL) cholesterol, but also to reduced production of other intermediates of the mevalonate pathway, including the non-sterol isoprenoids, farnesyl, pyrophosphate, and geranylgeranyl pyrophosphate (21). Farnesyl pyrophosphate and geranylgeranyl pyrophosphate are required for post-translational modification (iso-prenylation) and biological activity of a wide variety of cellular proteins, including the small guanosine triphosphatases Ras and Rho (22), which are strongly implicated in carcinogenesis (23, 24). Modulation of iso-prenylation appears to be a central mechanism through which statins exert their anti-proliferative and pro-apoptotic effects (23–25). Statins are known to exert a variety of effects, including having anti-cancer properties, in addition to their ability to lower cholesterol levels (43). Hence, repurposing statins in cancer therapeutics could be crucial for increasing the overall survival of patients (44). Dysregulation of the mevalonate pathway may be implicated in neoplastic transformation and tumor progression and this may partly explain the tumor-selective effects of statins (26, 27). A number of HMG-CoA reductase-independent mechanisms have also been proposed to account for the pleiotropic effects of statins, including antioxidant activity (28) and effects on cell adhesion (29, 30), inflammation (31, 32), immune regulation (33), and angiogenesis (34).

The type and dosage of statins have varied in retrospective observational studies (19, 20). Chemoprevention studies have used first- and second-generation statins more commonly and the mean dosage has been 30–40mg (35). The mean dosage was 30–40 mg when given along with NACTRT (20). Rosuvastatin is a third-generation statin with very minimal toxicity (36). Given its structural difference and hydrophilic nature, it provides better efficacy compared to other statins (42). The standard dose of rosuvastatin is 5-40mg. The 40mg dose is used only when the serum LDL goal is not reached by standard dosage, however, it can be associated with an increased risk of adverse events, especially in some subgroups such as Asian patients and women (37). Therefore, we proposed using a mean rosuvastatin dosage of 20 mg in the trial to have minimal side effects as we are using rosuvastatin as an adjunct to the chemotherapy.

Furthermore, to understand the molecular mechanism, it is crucial to investigate the effect of statins on the chromatin organizer Special AT-rich binding protein 1 (SATB1), a downstream target and master regulator of Wnt signaling (44, 49). It has been shown that SATB1 directly affects the onset and spread of various cancers such as colorectal (50) and breast cancer (51). Further, statins treatment is known to downregulate SATB1 in colorectal cancer cells (52) but the effect on its homolog SATB2 is not known. Therefore, examining the expression level of SATB proteins following statins administration along with NACTRT versus NACTRT alone would provide a comprehensive understanding of the probable molecular mechanism of action for the therapeutic effectiveness of rosuvastatin.

### 1.2 Study rationale

A pCR following NACTRT has been associated with decreased local recurrence and improved recurrence-free survival in rectal cancer. Statins have been shown to induce better pCR in combination with NACTRT in observational studies (39). This study aims to assess whether this observation is true through the conduct of a randomized study comparing treatment with statins along with NACTRT versus NACTRT alone (**Table 1A**).

**Table 1A:** Summarized study hypothesis and objectives.

	Phase II	Phase III
Hypothesis	The addition of Rosuvastatin to NACRT will improve the pathological complete response rate by 10% (from 15% to 25%)	The addition of Rosuvastatin to NACTRT will improve the 3-year disease-free survival by 7% (from 60% to 67%)
Primary objective	To determine the impact of rosuvastatin in improving the pCR rate in patients with localized rectal cancer undergoing neoadjuvant chemoradiotherapy	To determine the impact of rosuvastatin in improving 3-year DFS in patients with localized rectal cancer undergoing neoadjuvant chemoradiotherapy
Secondary objectives	•The frequency and severity of adverse events in the two arms as assessed by CTCAE v4.03 •To compare postoperative morbidity and mortality in the two arms •To identify other potential prognostic and predictive factors determining outcomes •To compare the disease-/progression-free survival in the two arms •To compare the overall survival in the two arms	•To compare the 5-year overall survival in the two arms •To compare the adverse events in the two arms •To identify other potential prognostic and predictive factors determining outcomes •To compare the patterns of recurrence in the two arms

**Figure 1:**
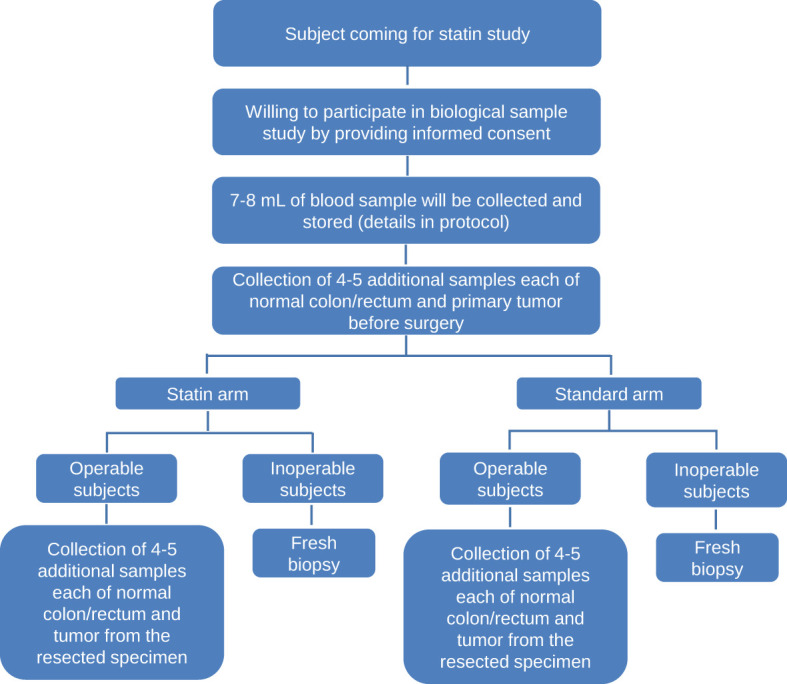
Flow chart depicting the molecular sub-study.

The molecular sub-study aimed to study the combined expression patterns of SATB family chromatin organizers which would provide a better understanding of colorectal cancer disease progression and would establish them as an unequivocal prognostic marker and a potential therapeutic marker for statins therapy (**Table 1B**, **Figure 1**). The complete details of the molecular sub-study is mentioned in **Appendix 5** in **Supplementary Material**.

**Table 1B:** Objectives for molecular sub-study.

Rationale	The molecular sub-study aims to study the combined expression patterns of SATB family chromatin organizers which would provide a better understanding of CRC disease progression and would establish them as an unequivocal prognostic marker and a potential therapeutic marker for statin therapy.
Objectives	•Evaluate expression and localization of SATB1 and SATB2 across paired human colon/rectum and tumor samples •Correlation of expression of SATB1 and SATB2 across different grades and stages of tumor samples towards understanding CRC progression •Understand the role of SATB proteins in tumor regeneration and disease relapse by validation of the association of its expression across differentiated and undifferentiated tumors •Correlation of expression of SATB proteins across colorectal cancer subtypes-Classical adenocarcinoma, mucinous adenocarcinoma, and signet ring pathology tumors

### 1.3 Study hypothesis and objective(s) in the phase II part

Hypothesis:

The addition of rosuvastatin to NACTRT will improve the pCR rate by 10% (from 15% to 25%).

Primary objective:

To determine the impact of rosuvastatin in improving pCR in patients with locally advanced rectal cancer undergoing NACTRT.

Secondary objectives:

- To compare the frequency and severity of adverse events in the two arms.
- To compare post-operative morbidity and mortality in the two arms.
- To identify other potential prognostic and predictive factors determining outcomes.
- To compare the DFS in the two arms.
- To compare the OS in the two arms.
- Evaluate expression and localization of SATB1 and SATB2 across paired human colon/rectum and tumor samples.
- Correlation of expression of SATB1 and SATB2 across different grades and stages of tumor samples towards understanding CRC progression.
- Understand the role of SATB proteins in tumor regeneration and disease relapse by validation of the association of its expression across differentiated and undifferentiated tumors.
- Correlation of expression of SATB proteins across colorectal cancer subtypes-classical adenocarcinoma, mucinous adenocarcinoma, and signet ring pathology tumors.

If the phase 2 study has positive results, we plan to proceed to a phase 3 study.

### 1.4 Study hypothesis and objective(s) in the phase III part

Hypothesis:

The addition of rosuvastatin to NACTRT will improve the 3-year DFS by 7% (from 60% to 67%).

Primary Objective:

To determine the impact of rosuvastatin in improving 3-year DFS in patients with locally advanced rectal cancer undergoing NACTRT.

Secondary objectives:

- To compare the 5-year OS in the two arms.
- To compare the adverse events in the two arms.
- To identify other potential prognostic and predictive factors determining outcomes.
- To compare the patterns of recurrence in the two arms.

## 2 Methods

### 2.1 Study design

The study is a prospective, randomized, open-label phase II/III study.

For the phase II part of the study, eligible patients will be randomized 1:1 to the statin + NACTRT arm or the only NACTRT (standard) arm. The statin arm will receive rosuvastatin 20 mg for 12–16 weeks. Pathological response would be assessed following surgery.

A follow-on phase III trial with 3-year DFS as the endpoint will be planned depending on how the statin arm fares in terms of feasibility, safety, and efficacy.

### 2.2 Inclusion and exclusion criteria

### 2.3 Detailed methodology

#### 2.3.1 Screening

All patients diagnosed as having rectal cancer will undergo a standard staging workup that includes but may not be limited to a colonoscopy, biopsy, pelvis MRI, and contrast-enhanced computed tomography (CECT) of the thorax and abdomen, carcino embryonic antigen (CEA), complete blood count and biochemistry tests [random blood sugar (RBS), liver function test (LFT), and renal function test (RFT)]. This is part of the standard care. Treatment decisions are taken by a multi-disciplinary tumor board (MDTB). Previously untreated rectal cancer patients who are planned for NACTRT will be screened for the study and an anonymized screening log will be maintained.

#### 2.3.2 Enrolment in the study/consent

All patients eligible for study participation who satisfy the inclusion/exclusion criteria (**Table 2**, **Appendix 4** in **Supplementary Material**) will be enrolled after an informed consent form (ICF), provided by a study team member, is signed. An enrolment log will be maintained and a study ID will be allocated to each patient at study entry.

**Table 2:** Inclusion and exclusion criteria.

Inclusion criteria	Exclusion criteria
•Age between 18–70 years •Subjects willing to sign the informed consent •Eastern Cooperative Oncology Group (ECOG) performance status 0 to 2 •Histologic diagnosis of rectal adenocarcinoma •Clinical and radiological T2–4 (any) N (any) M0 •Fit to receive neoadjuvant chemoradiotherapy •Absence of colorectal synchronous primary •No prior rectal cancer treatment received	•Patients with proven metastatic disease •History of Crohn's disease or ulcerative colitis •Inherited polyposis syndromes •Ongoing statin or aspirin therapy for cardiovascular disease •Pregnant or nursing women •Subject not willing to provide informed consent •Comorbidities precluding statins and neo-adjuvant therapy including but not limited to Hepatitis *, acute or chronic kidney disease* •Prior anti-neoplastic therapy •Patients already taking the following drugs: cyclosporine, gemfibrozil, atazanavir/ritonavir, lopinavir/ritonavir, tipranavir/ritonavir or simeprevir, eltrombopag, dronedarone, itraconazole, fluconazole or ketoconazole; coumarin anticoagulants; lipid-lowering therapies: fibrates or lipid-modifying doses (greater than or equal to 1g/day) of niacin; aluminum and magnesium hydroxide combination; antacid; and erythromycin. *Hepatitis will be defined as sustained elevation of transaminases (two readings 5–7 days apart) ≥3X upper limit of normal. *Kidney disease will be defined as elevated creatinine levels above the upper limit of normal

#### 2.3.3 Randomization

Patients will be randomized in a 1:1 ratio using block randomization to rosuvastatin and NACTRT or NACTRT alone. An independent statistician will generate the randomization code and independent research staff will inform the study team of the allocation. A randomization log will be maintained by the study team.

#### 2.3.4 Treatment

##### 2.3.4.1 Standard of care

Both the study groups will receive NACTRT as per the standard of care. Furthermore, 45–50 Gy of radiotherapy will be administered to the pelvis for period of 5–6 weeks. Concurrent tablets of capecitabine of 825mg/m2 will be administered orally twice daily from day 1 to day 35 along with radiotherapy.

##### 2.3.4.2 Study intervention

**Figure 2:**
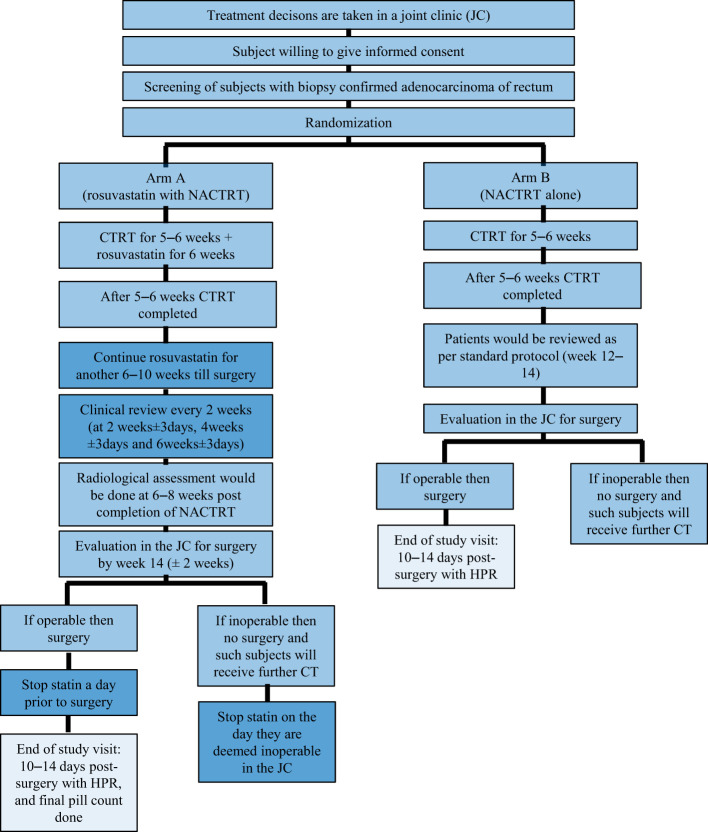
CONSORT flow diagram depicting the trial schema for treatment decisions. .

Patients in the study arm will receive 20 mg rosuvastatin orally once daily along with NACTRT for 6 weeks followed by rosuvastatin alone for another 6–10 weeks until surgery (12–16 weeks of statin therapy until surgery or confirmation of ineligibility for surgery). Patients in the control arm will receive only NACTRT as described above, which is the standard of care (**Figure 2**).

##### 2.3.4.3 Drug dispensing

Rosuvastatin will be dispensed at randomization and at week 7. Patients who have logistical issues will be dispensed 16 weeks of rosuvastatin prior to starting NACTRT. Patients will be asked to provide used and unused packs of the drug at each visit and a pill count will be conducted and details noted in the case record form (CRF) and drug dispensing logs.

#### 2.3.5 Follow-up procedures

After commencing the statin, the patients in the intervention arm will be reviewed every 2 weeks on NACRT (at 2 weeks ± 3 days, 4 weeks ± 3 days, and 6 weeks ± 3 days) either in person in the clinic or telephonically if they cannot come to the clinic due to unavoidable circumstances. Patients in the standard arm on NACTRT will be reviewed as per standard protocol. Patients in both arms will be reviewed at 6 ± 2 weeks after completion of NACTRT (weeks 12–14). At this visit, all patients will undergo restaging as per routine practice and will be evaluated by the MDTB for surgery by week 14 (± 2 weeks). Patients in both arms will be operated on if deemed to have operable disease.

#### 2.3.6 Assessment of response

Clinical and radiological assessment will be done at 6–8 weeks after completion of NACTRT. Eligible patients will undergo standard surgery (anterior resection, abdominoperineal resection, inter-sphincteric resection, or multi-visceral resection) according to the level and extent of growth. If the reassessment MRI at 12–14 weeks after completion of NACTRT shows inoperable disease, then the patient will be deemed a non-responder and analyzed as part of the non-pCR group. These patients, if on the rosuvastatin arm, will stop the statin on the day they are deemed inoperable by the MDTB. These patients will receive further chemotherapy to downstage the disease as per the current standard of care. A histopathological examination will be conducted for the patients operated on and the pathological response rate and tumor regression grade (TRG) will be judged according to the American Joint Committee on Cancer (AJCC) (**Appendix 1** in **Supplementary Material**). A pCR is defined as no residual cancer found on histological examination of the TME specimen (Grade 0 AJCC or Grade 1 Mandard’s). The pathologist assessing the specimen will be blinded to the treatment that the patient received.

Following surgery and the availability of the histopathology report, the patient would have completed the active phase of the study and will be followed up according to standard follow-up protocol. Survival details will be collected at each follow-up visit or telephonically if the patient misses a visit for any reason.

#### 2.3.7 End of study visit

Patients will receive rosuvastatin for 12–16 weeks, with the treatment being discontinued 1 day prior to surgery for operable patients. The final study visit for these patients will coincide with their MDTB appointment, where the final surgical histopathology report (approximately 10–14 days post-surgery) will be reviewed. For patients deemed inoperable, the study will conclude on the day the decision of inoperability is made. At this visit, a final safety follow-up and pill count will be conducted. Following this, there will be no further active intervention, and patients will be monitored for survival. No protocol-mandated imaging or re-evaluation is planned; patients will be followed according to standard surveillance guidelines for rectal cancer patients who have undergone multimodality treatment.

#### 2.3.8 Study specific investigations

All investigations will be done as per the current standard of care. There will be no additional planned blood tests or imaging done for the purpose of the study. In patients who agree to participate in the molecular sub-study, the baseline samples will be taken at the time of the baseline endoscopy and post-treatment samples for the operated patients will be from the resected specimen. Post-treatment samples for patients who are not operated on (approximately 30% of those receiving NACTRT) will be collected by conducting a repeat limited sigmoidoscopy. The patients will be reimbursed for this and the same is mentioned in the ICF as well. The follow-up will be as per the prevailing standard of care.

The schedule of events are depicted in **Table 3**.

**Table 3:** Schedule of events.

	Baseline	Randomization	Week 2 visit ±3 days	Week 4 visit ± 3days	Week 6 and 7 visit ±3 days	Week 12 visit ±3 Days	Week 14 to 16 visit ±3 days
Informed Consent	✓						
Screening of patients with biopsy-confirmed adenocarcinoma of the rectum	✓						
Collection of four additional tissue samples for molecular sub-study	✓						
Collection of 7–8 ml of blood	✓						
Randomization		✓					
Both arms NACTRT			✓	✓	✓		
Statin dispensed		✓			✓		**
Statin pill counts			✓	✓	✓		✓
Adverse event/serious adverse event reporting			✓	✓	✓	✓	✓
Clinical review			✓	✓	✓	✓	✓
Radiological review						✓*	✓*
MDTB decision for surgery							✓
Collection of four additional tissue sample post-surgery							✓#

*Radiological assessment will be done at 6–8 weeks after completion of NACTRT.

**Rosuvastatin will be stopped a day prior to surgery (operable patients) or on the day deemed inoperable (inoperable patients).

#Paired tissue samples will be collected from patients who are operated on via the resected specimen (post-treatment sample) or fresh biopsies for patients who are not operated on.

#### 2.3.9 Withdrawal/stopping criteria

The patient will be withdrawn from the study if:

- The patient develops unacceptable toxicity.
- If the patient wishes to withdraw from participation.
- There is a need for a dose reduction of more than 1 level or discontinuation of the study drug.
- The patient does not comply with the protocol.

Patients who are not operated on despite a response will not be analyzed in the phase II study but will be followed up for survival and included in the analysis of the phase III study if it is conducted.

#### 2.3.10 Adverse events

At each visit, the investigator will evaluate the patient to determine whether any adverse events (AEs) have occurred. AEs may be directly observed, reported spontaneously by the patient, or questioned by a study team member patient at each study visit in the clinic or on the telephone. The NCI Common Terminology Criteria for Adverse Events version 4 (NCI CTCAE v4.03) will be used to classify and grade the intensity of adverse events during and after treatment. A serious adverse event (SAE) or AE is defined as the diagnosis or a sign or symptom as applicable as per the CTCAEv4.03. All events, whether related or not, will be recorded and graded and the worst toxicity will be recorded. All laboratory values will be evaluated by the investigator as to their clinical significance. All abnormal laboratory values considered clinically significant by the investigator will be recorded as an AE.

All AEs/SAEs will be followed up till resolution or stabilization. In case of unresolved AEs, including significant abnormal laboratory values at the end of treatment assessment, these events will be followed up until resolution or until they become clinically not relevant. Pre-planned procedures or hospitalization for pre-existing conditions that do not worsen in severity will not be reported as SAEs. Progressive disease or death due to progressive disease will not be reported as an SAE. AEs/SAEs occurring after the end of the study period will not be reported.

The definitions of AEs/SAEs and reporting are outlined in **Appendix 2** in **Supplementary Material**.

### 2.4 Sample size and duration of the study

Each year, approximately 180–200 rectal cancer patients receive NACTRT at our hospital, with approximately 70% undergoing surgery and 15%–20% developing progressive disease. The current pCR rate at our hospital following NACTRT is approximately 15%, while the literature indicates a pCR rate of 25%–30% with statin therapy. We hypothesize that rosuvastatin can increase the pCR rate by 10%, from 15% to 25%. To detect a 10% difference in pCR rates, with a two-sided alpha of 0.10 and 80% power, a sample size of 288 patients would be required. Factoring in a 10% attrition rate, the total sample size increases to 316. Approximately 500–600 patients will need to be screened to enroll 316 participants. It is estimated that accrual and analysis will take approximately 3 years to complete.

A follow-on phase III trial is also will be planned depending on the success of the phase II study, which will compare the 3-year DFS in the two arms. All the patients in the phase II study will be included in the survival statistics in the follow-on phase III study which will be statistically powered for survival. Assuming that the addition of rosuvastatin to NACTRT will improve the 3-year DFS from 60% to 67%, our calculated sample size will be 1,135 subjects (567 in the control group and 568 in the treatment group) (a two-sided log-rank test with 80.0% power at a 0.050 significance level). This assumes a uniform accrual pattern across time periods and 10% dropout rate. If we move on to the phase III study, another 4 years will be required for accrual. The total duration will be 7 years for accrual and 3 years for follow up.

### 2.5 Statistical consideration

A) The primary efficacy variable is a pathological complete response which is defined as the absence of microscopic tumors in the resected specimens in patients who undergo surgery following neoadjuvant chemoradiotherapy.

B) The secondary efficacy variables are toxicity and 3-year DFS.

C) Toxicity will be assessed as detailed below. The NCI Common Terminology Criteria for Adverse Events version 4 (NCI CTCAE v4.03) will be used to classify and grade the intensity of adverse events during and after chemo-radiotherapy. The worst grade of each toxicity episode will be recorded. All events regardless of attribution will be graded so as to ensure objectivity in reporting as per accepted international guidelines. The details will be entered in the dataset.

D) Any adverse event that occurs from the day of randomization until the end of study visit will be defined as an acute adverse event. Events that occur thereafter will be defined as chronic adverse events. Both acute and chronic adverse events will be recorded however only events happening till 30 days after surgery will be reported and not beyond.

E) Postoperative morbidity and mortality will be any event occurring from the day of surgery until 30 days.

F) Disease-free survival is defined as the time from the date of randomization until the date of disease recurrence or until death from any cause.

G) Progression-free survival (PFS) is defined as the time from the date of randomization until the date of disease recurrence or until death from any cause. If there is a clinical suspicion of progression, further workup and management will be as per the institutional standard. Progression will be assessed by the investigator using appropriate workup and investigations, as per routine care.

H) Overall survival is defined as the time from the date of randomization until the date of death from any cause.

### 2.6 Statistical analysis

Quantitative data will be presented as mean ± SD and categorical variables as frequencies within each cohort. The comparison of the statin and non-statin groups will be done using Fisher’s exact test or a χ2 test for categorical variables, and a Wilcoxon rank sum test for quantitative variables. Univariate analysis of significant predictors of an AJCC grade 0 response to NACTRT will be performed by a χ2 or Fisher’s exact test for categorical variables and logistic regression for quantitative variables. Multivariate analysis of statin use as a predictor of AJCC grade 0 responses to NACTRT will be done using multivariable logistic regression models. A Kaplan–Meier survival analysis will be used to determine the effect of statin use and AJCC TRG score on overall survival and disease-free survival. Cox proportional hazard models will be used to determine whether statin use was a significant predictor of recurrence and survival outcomes after adjusting for predicted covariates. All secondary endpoint comparisons will be made using the two-sided log-rank test with a 0.05 significance level. Assuming that OS demonstrates significance in the phase II study, the same criteria would be used for the analysis of PFS. For potential prognostic and predictive factors, multivariate analysis would be performed based on gender, metastasis occurrence from accrual to follow-up timepoint, histology subtype (diffuse, intestinal), and measurable disease (yes, no).

### 2.7 Ethics

Ethics approval statement:

This study (Project no. 3033) was approved by Institutional Ethics Committee II of Tata Memorial Hospital on 1^st^ September 2018.

Clinical trial registration: Registered at the Clinical Trials Registry of India. CTRI/2018/11/016459

## 3 Study drug-related information

### 3.1 Dosing schedule (dose, frequency, and duration of the experimental treatment)

A 20 mg rosuvastatin tablet will be administered orally as a single dose 1 hour after food at night for 12–16 weeks.

### 3.2 Rosuvastatin administration

A member of the study team will prescribe the drug and give instructions about its usage. Regular intake of the drug will be confirmed by weekly telephonic calls with the study coordinator. Patients will be followed up at regular intervals according to the study protocol unless any adverse events occur, which will be managed immediately. Drug compliance will be monitored and noted in the CRF.

The details of rosuvastatin drug toxicity, dose modification, possible drug interactions, concomitant therapy in the study, and dosing delays/dose modifications are mentioned in **Appendix 3** in **Supplementary Material**.

## 4 Study monitoring and supervision

The study has been monitored thrice by the National Cancer Grid contract research organization (NCG-CRO) and once by the institutional research review committee. All the monitoring reports have been forwarded to the institutional ethics committee (IEC).

## 5 Limitations

If any adverse effects of rosuvastatin are reported, they will be managed according to the National Lipid Association (NLA) Statin Safety Task Force guidelines (40, 41). According to these, monitoring CK levels is recommended only for symptomatic patients. The first-level dose reduction will be to 10 mg. If another level of reduction is required, the drug will be discontinued and the patient will be withdrawn from the study. Moreover, there are studies that have reported the effect of alcohol consumption, smoking, and body mass index on treatment response to statins (47, 48). However, the status of these was not considered in our exclusion criteria for the study. Further, the proposed study protocol has a short follow-up duration for phase II which could be improved if the study is extended for phase III.

## 6 Discussion

The addition of adjuncts to NACTRT has the potential to improve outcomes by increasing the likelihood of achieving a pCR in rectal cancer patients. Enhancing pCR rates adds significant value to treatment, as more patients achieving a pCR may enable the adoption of a “watch and wait’” strategy for a select subgroup of CRC patients. Tumor downstaging could also facilitate local excision without compromising survival outcomes. Statins, a low-cost intervention with a well-established safety profile from decades of use, offer a promising option in this context. If this study confirms their efficacy and safety, statins could serve as a valuable adjunct for rectal cancer patients receiving NACTRT.

Future research should prioritize the use of patient-derived models and large-scale phase III randomized clinical trials involving cancer patients. These trials are critical to validating predictive biomarkers and understanding the precise role of statins in cancer prevention and treatment. Such investigations will shed light on the molecular mechanisms underlying the tumor-suppressive properties of statins and help establish the specificity of their anti-cancer effects.

## 7 Conclusion

The addition of rosuvastatin to NACTRT has the potential to enhance oncological outcomes by increasing the likelihood of achieving pCRs in rectal cancer patients. A higher pCR rate could enable the implementation of a “watch and wait” strategy for a select subgroup of patients. This study aims to determine whether the observed link between statin use and improved pCR rates in observational studies represents an association or causation. Additionally, the molecular sub-study will provide valuable insights into the potential mechanisms of action of statins and aid in identifying prognostic markers to further understand their therapeutic impact.

## Data Availability

The original contributions presented in the study are included in the article/supplementary material. Further inquiries can be directed to the corresponding author.
