# Supplementary material for: A randomized phase II/III trial of rosuvastatin with neoadjuvant chemo-radiation in patients with locally advanced rectal cancer
Source: Front Oncol. 2025 Mar 19;15:1450602. doi: 10.3389/fonc.2025.1450602 (PMC11961435; doi:10.3389/fonc.2025.1450602)
Supplement: Supplementary file 1 [file DataSheet1.pdf]

Appendices

Appendix 1: Tumor regression grade (TRG) systems

Table A:AJCC Tumor regression grade (TRG) system

TRG 0	No residual tumor cells
TRG 1	Single cell or small group of cells
TRG 2	Residual cancer with desmoplastic response
TRG 3	Minimal evidence of tumor response

Table B: Mandard's Tumor regression grade (TRG) system

Complete regression (pathological complete response/pCR)	No residual cancer cells (TRG1)
Near complete regression	Rare residual cancer cells-(TRG 2)
Moderate regression	Predominant fibrosis with increased Number of residual cancer cells (TRG 3)
Minimal regression	Residual cancer out growing fibrosis (TRG 4)
No regression	No regressive change (TRG 5)

Appendix 2: The definitions and reporting of AEs/ SAEs

1. Definitions

An **Adverse Event (AE)** is any untoward medical occurrence in a patient or clinical investigational subject administered a pharmaceutical product and which does not necessarily have a causal relationship with this treatment. An AE can therefore be any

unfavourable or unintended sign (including an abnormal laboratory finding), symptom, or disease temporally associated with the use of a medicinal investigational product, whether or not considered related to the medicinal product (see below).

Adverse events include the following:

1. All suspected adverse drug or device reactions
2. All reactions from drug or device–over dose, abuse, withdrawal, sensitivity, toxicity or failure of expected pharmacological action (if appropriate).
3. Apparently unrelated illnesses, including the worsening (severity, frequency) of pre-existing illnesses
4. Injury or accidents.
5. Abnormalities in physiological testing or physical examination that require clinical intervention or further investigation (beyond ordering a repeat examination)
6. Laboratory abnormalities that require clinical intervention or further investigation (beyond ordering a laboratory test).
7. Any untoward event that occurs after the protocol-specified reporting period which the Investigator believes may be related to the drug or device.

AEs will be documented but will not be reported unless they meet SAE criteria.

A serious adverse event (SAE) is any untoward medical occurrence that at any dose that results in

1. Death,
2. Is life-threatening (i.e. the subject is at risk of death at the time of the event),
3. Requires in patient hospitalization or prolongation of existing hospitalization,
4. Results in persistent or significant disability or incapacity,
5. Is a congenital anomaly/birth defect,
6. Other important medical events which, in the opinion of the investigator, are likely to become serious if untreated, or as defined in the protocol.
7. Important medical events which may not be immediately life-threatening or result in death or hospitalization but which may jeopardize the patient or may require

intervention to prevent one of the listed outcomes in the definition above should also be considered serious.

Causality assessment: An event is causally related if there is a reasonable possibility that the drug caused it (evidence to suggest a causal relationship between the drug and the event).

Accordingly, Rosuvastatin, Capecitabine, radiation therapy or surgery will beat tributed a degree of causality from one of the following codes:

- Unrelated
- Unlikely to be related
- Possibly related
- Probably related
- Definitely related

Hospitalization or death as a result of or related to disease progression will not be reported as SAEs: AEs/ SAEs occurring after the end of study visit will not be reported.

2 SAE reporting:

All adverse events, which occur whilst the participant is enrolled on the trial, will be recorded in the patients' medical records and on the CRF and reported as per current regulatory requirements (24 hrs for serious adverse events) in the format approved by the Ethics committee. The Common Terminology Criteria for Adverse Events (CTCAE version 4.03) will be used to grade the severity of any event.

Appendix 3: The details of Rosuvastatin drug toxicity, dose modification, possible drug interactions, concomitant therapy on the study, dosing delays/dose modifications

3.1 Rosuvastatin drug toxicity and dose modification

Contraindications to Rosuvastatin therapy:

The Rosuvastatin label mentions the following contraindications and these patients will be excluded from trial participation.

• Known hypersensitivity to product components (statin)

• Active liver disease, which may include unexplained persistent elevations in hepatic transaminase levels

• Women who are pregnant or may become pregnant

• Nursing mothers

Statin monotherapy:

Rosuvastatin is a 3rd generation statin has high potency and efficacy and thus termed as super statins [1]. It owes remarkable potency and efficacy due to its fluorinated phenyl group and hydrophilic methane sulphonamide group in addition to the common dihydroxy heptanoic acid side chain. Its unique chemical structure enables multiple and strong binding with HMG-CoA reductase enzyme [2].

Data from the clinical development program have shown that rosuvastatin is well tolerated regardless of age, gender, ethnicity, or presence of comorbidities or concomitant medications [3].The most commonly reported adverse events reported with rosuvastatin are pharyngitis, headache, diarrhoea, dyspepsia and nausea[4]. Other rare side effects are myalgia, myopathy, reversible proteinuria and transient elevation of liver enzymes [5]. Evidence of muscle toxicity with the 5–40 mg doses is minimal. There is a very low incidence of myopathy (<0.1%) and rhabdomyolysis (<0.01%). Rosuvastatin should be prescribed with caution in patients predisposed to myopathy/rhabdomyolysis [6]. Rosuvastatin is well tolerated from a renal perspective. Proteinuria usually transient, tubular rather than glomerular, and not associated with acute or progressive renal disease [7]. Elevations in liver transaminases occur in frequently with statin use. In clinical trials, clinically significant increases in serum liver transaminases (alanine aminotransferase [ALT] >3- times ULN on more than two consecutive occasions) occurred in 0.2% of patients and were not associated with clinical evidence of liver dysfunction or failure [8].

Statins in combination with chemo/radiotherapy:

In one study, in patients treated with Simvastatin + XeLIri/ FOLFIRI- Grade 3 or higher grade nausea and anorexia were noted slightly more in patients with simvastatin arm compared to placebo arm (4.5 vs 0.7%, 3.0 vs 0% respectively) [9]. No other grade 3 or higher toxicity was reported. In retrospective studies looking at patients with Rectal cancer on statins who were treated with NACTRT, no increased toxicity was seen [10,11].

Management of statin-associated adverse effects

Adverse effects of Rosuvastatin would be managed according to the National Lipid Association (NLA)Statin Safety Task Force guidelines [12,13]. According to these, monitoring CK levels is recommended only for symptomatic patients. Also, routine monitoring of liver function, renal function, or cognitive function is not recommended.

First level dose reduction will be to 10 mg. If another level of reduction is required, the drug will be discontinued and the patient will be withdrawn from the study.

3.2 Possible drug interactions:

Rosuvastatin has low drug interaction potential due to its hydrophilic nature, which avoids biotransformation for conversion into water-soluble intermediates for elimination. Another reason being drug less likely under goes little or no metabolism by the CYP3A4 isoenzyme. This has been confirmed through specific interaction studies.

The concomitant medications will be avoided while patients are on the study. Patients already taking those drugs will be excluded from the study. (Appendix 4).

3.3 Concomitant therapy on the study: Rosuvastatin would be given along with NACTRT (Tablet capecitabine and external beam radiation).There are no drug interactions or adverse effects documented for these. Patients will receive appropriate antiemetics, nutritional support and hydration as well as other supportive care medications as per current standard of care while on NACTRT.

3.4 Dosing Delays/Dose Modifications: Current guidelines suggest dose reduction and halting of statin therapy only in extreme conditions as explained above. One level dose reduction will be to a dose of 10mg. If another level of reduction is required then the patient will be withdrawn from the study.

3.5 References

1. Kapur NK, Musunuru K. Clinical efficacy and safety of statins in managing cardiovascular risk. Vasc Health Risk Manag. 2008;4(2):341-53.

2. Holdgate GA, Ward WH, McTaggart F. Molecular mechanism for inhibition of 3-hydroxy-3-methylglutaryl CoA (HMG-CoA) reductase by rosuvastatin. Biochem Soc Trans. 2003;31(Pt 3):528-31

3. Grundy SM. The issue of statin safety: where do we stand? Circulation. 2005; 111(23):3016-9.

4. Martin PD, Mitchell PD, Schneck DW. Pharmacodynamic effects and pharmacokinetics of a new HMG-CoA reductase inhibitor, rosuvastatin, after morning or evening administration in healthy volunteers. Br J Clin Pharmacol. 2002;54(5):472-7.

5. Tripathi, Sneha, Ekta Gupta, and Sanjeev Galande. Statins as anti-tumor agents: A paradigm for repurposed drugs. Cancer Reports 2024; 7(5): e2078.

6. Brewer HB Jr. Benefit-risk assessment of Rosuvastatin 10 to 40 milligrams. Am J Cardiol. 2003;92(4B):23K-29K.

7. Vidt DG, Cressman MD, Harris S, Pears JS, Hutchinson HG. Rosuvastatin-induced arrest in progression of renal disease. Cardiology. 2004;102(1):52-60.

8. Shepherd J, Hunninghake DB, Stein EA, Kastelein JJ, Harris S, Pears J, Hutchinson HG. Safety of rosuvastatin. Am J Cardiol. 2004; 94(7):882-8.

9. Lim SH, Kim TW, Hong YS, Han SW, Lee KH, Kang HJ, Hwang IG et al. A randomised, double-blind, placebo-controlled multi-centre phase III trial of XELIRI/FOLFIRI plus simvastatin for patients with metastatic colorectal cancer. British J Cancer 2015; 113(10): 1421-1426.

10. Katz MS, Minsky BD, Saltz LB, Riedel E, Chessin DB, Guillem JG. Association of statin use with a pathologic complete response to neoadjuvant chemoradiation for rectal cancer. Int J Radiat Oncol Biol Phys. 2005; 62(5):1363-70.

11. Mace AG, Gantt GA, Skacel M, Pai R, Hammel JP, Kalady MF. Statin therapy is associated with improved pathologic response to neoadjuvant chemoradiation in rectal cancer. Dis Colon Rectum. 2013; 56(11):1217-27.

12. McKenney JM, Davidson MH, Jacobson TA, Guyton JR; National Lipid Association Statin Safety Assessment Task Force. Final conclusions and recommendations of the National Lipid Association Statin Safety Assessment Task Force. Am J Cardiol. 2006; 97(8A):89C-94C.

13. Jacobson TA. NLA Task Force on Statin Safety--2014 update. J Clin Lipidol. 2014; 8(3 Suppl):S1-4.

Appendix 4: List of prohibited drugs :

Cyclosporine	Gemfibrozil	Anystatin	Aspirin
Other lipid-lowering therapies: fibrates or lipid modifying doses (greater than or equal to 1 g/day) of niacin	Atazanavir/ritonavir, lopinavir/ritonavir, Tipranavir/ritonavir or simeprevir,	Itraconazole, Fluconazole or Ketoconazole	Aluminium and magnesium hydroxide combination antacid
Eltrombopag	Coumarin anticoagulants	Dronedarone	Erythromycin

Appendix 5: Molecular sub study: Understanding role of SATB (1/2) proteins in regulation of colorectal cancer progression

5.1 Introduction:

Over recent decades, the molecular basis of colorectal cancer (CRC) pathogenesis has been extensively explored to identify potential molecular markers and therapeutic targets. In this regard, the special AT-rich sequence-binding proteins 1 and 2 (SATB1/2) have emerged as global regulators, with their roles as chromatin organizers being implicated in CRC development and progression [1]. SATB1 functions as a genome organizer, orchestrating epigenetic changes in specialized genomic regions and regulating the expression of numerous genes, while SATB2, a close homolog, exhibits similar

functions [2]. The differing expression patterns of SATB1 mRNA and protein between colorectal cancer and normal tissues highlight its potential as a promising target for cancer therapy [3]. SATB1 has been identified as a key global regulator of tumor growth, metastasis, and angiogenesis in CRC progression [4]. On the other hand, SATB2 serves as a sensitive marker to distinguish CRC from other cancers, although reduced SATB2 expression in CRC is associated with poor prognosis [5].

Statins, widely recognized for their cholesterol-lowering properties, have also been shown to significantly improve patient survival in CRC treatment [6]. Previous studies indicated that statin therapy reduces SATB1 levels, which could contribute to simvastatin's therapeutic effectiveness in CRC cells [7]. In this context, evaluating SATB chromatin organizer expression in patient samples undergoing statin therapy may provide insights into the mechanisms underlying their therapeutic impact.

5.2 Sample size:

All 316 patients will undergo testing at baseline and post treatment at 80% power and alpha error 0.05 (5%) and assuming a standard deviation of 2.2 and a sample quality error of 20%.

5.3 Methodology:

i. Tissue collection and characterization

All patients accrued in the statin study will be given the option for participating in the molecular sub-study and if willing, will sign the molecular sub part of the informed consent document. Paired tissue samples of normal rectum and tumor (4 pieces each) before and after statin treatment will be collected as fresh biopsies in the endoscopy room ('pre-treatment' sample) or from the resected specimen (for operated patients, 'post-treatment' sample) or fresh biopsies (for patients who do not get operated) and processed as described below.

The samples will be collected aseptically in a sterile container. The tumor tissue will be divided for RNA, DNA and protein analysis, and for routine histopathology and IHC studies. Two pieces will be stored frozen at -80˚C while the two pieces will be stored 4˚C (on ice or in a cooler) in a sterile container containing DMEM (cell culture medium).

Tumor samples will be processed for H&E and assesses for grade, differentiation and presence of signet ring or mucinous morphology.

ii. Construction of Tissue Microarray (TMA)

Tissue microarray blocks will be made from the donor blocks manually in the Molecular Pathology and Translational Laboratory. A printed layout sheet will be designed in which the details of the donor blocks (Pathology numbers) taken for TMA will be entered. The starting point will be marked to locate the correct orientation on the TMA block. The TMA sections will be stained with H & E to define the representative tumor tissue. TMA with 20 cores per block will be constructed and three cores per tumor sample will be taken.

iii. Immunohistochemistry (IHC)

SATB1 and SATB2 will be performed using standardized antibodies as follows: Anti-SATB1antibody [EPR3895] (ab92307), Anti-SATB2 antibody (ab34735) with appropriate controls.

IHC staining of TMA sections will be performed using standard protocol. To state briefly, the TMA sections will be deparaffinized in three changes of xylene of 5 min each, and re-hydration in three changes of descending grades of alcohol of 5min each. Endogenous peroxidases blocking/ inactivation by methanol/H2O2 bath for 15 min will be followed by the antigen retrieval by heat induced epitope retrieval using10mM Tris EDTA buffer (pH 9.0) followed by one wash with TBS (pH 7.4).

IHC will be done using primary antibodies against SATB1 and SATB2 with controls. Secondary detection kit using Envision polymer kit followed by chromogen development with diaminobenzidine will be done. IHC will be read by pathologist based on semi quantitative assessment of tumor staining by estimating the percentage tumor positivity and the intensity of staining.

iv. Immunoblotting for SATB chromatin organizers

Total cellular protein would be extracted from the tissue samples in RIPA lysis buffer and estimated using BCA protein estimation kit (Thermo-scientific). Protein would be denatured by heating at 95˚C for 5 minutes resolved on 10-15% denaturing SDS-PAGE and transferred onto a PVDF membrane and would be incubated overnight with primary antibody (SATB1 and SATB2). After 3 buffer washes, the blot would be exposed to HRP-

conjugated secondary antibody for 3 hours, washed and developed using Pierce Super Signal West Pico chemiluminescence substrate (Pierce, USA).

v. RNA isolation and cDNA Synthesis

RNA would be isolated from the tissue samples using Trizol reagent (Invitrogen, Waltham, MA, USA). Two micrograms of RNA would be used for first strand cDNA synthesis using High capacity cDNA synthesis kit (Invitrogen). The cDNA would be then used for quantitative PCR (qRT- PCR).

vi. Quantitative real-time PCR for SATB chromatin organizers

qRT- PCR analyses with SATB1, SATB2 specific gene primers would be carried out with Step one plus in 96 well plate format using SYBRGreenMix (Life Technologies). Changes in threshold cycle (CT) values would be calculated as: Δ CT = CT (test) − CT (control); fold difference would be calculated as: fold difference = $2-\Delta$ (Δ CT). GAPDH expression would be used for normalization; non-template controls would account for possible contaminating DNA in reaction mixtures.

vii. Generation of Patient derived xenografts (PDX)

Tumor tissues would be digested to prepare single cell suspension. $5x10^6$ tumor cells would be injected subcutaneously in NOD/SCID mice. Mice would be observed for formation of patient derived xenografts. Surgically excised tumors would then be analyzed for expression of SATB family chromatin organizers.

viii. Cell line establishment

Tumor tissues would be digested to prepare single cell suspension, and further would be cultured to generate rectal cancer cell lines.

5.4 References:

1. Mir R, Pradhan SJ, Patil P, Mulherkar R and Galande S. Wnt/β-catenin signalling regulated SATB1 promotes colorectal cancer tumorigenesis and progression. Oncogene. 2015; 35:1679-1691.
2. Panchal O, Wichmann G, Grenman R, Eckhardt L, Kunz-Schughart LA, Franke H, Dietz A, and Aigner A. SATB1 as oncogenic driver and potential therapeutic target in

head & neck squamous cell carcinoma (HNSCC). Scientific Reports 2020; 10 (1): 8615.

3. Mir R, Pradhan SJ, Galande S. Chromatin organizer SATB1 as a novel molecular target for cancer therapy. Curr Drug Targets. 2012;13:1603-1615.
4. Naik R, and Galande S. SATB family chromatin organizers as master regulators of tumor progression. Oncogene 38, no. 12 (2019): 1989-2004.
5. Zhang, Y-J, Chen J-W, He X-S, Zhang H-Z, Ling Y-H, Wen J-H, Deng W-H et al. "SATB2 is a promising biomarker for identifying a colorectal origin for liver metastatic adenocarcinomas." EBioMedicine 28 (2018): 62-69.
6. Tripathi S, Gupta E, and Galande S. Statins as anti-tumor agents: A paradigm for repurposed drugs. Cancer Reports 7, no. 5 (2024): e2078.
7. Reddy CNL, Vaijayanti VN, Notani D, Galande S, Kotamraju S. Down-regulation of the global regulator SATB1 by statins in COLO205 colon cancer cells. Mol. Med. Rep. 2010; 3:857-861.
